# The emerging role of long noncoding RNA RMRP in cancer development and targeted therapy

**DOI:** 10.20892/j.issn.2095-3941.2021.0577

**Published:** 2022-01-26

**Authors:** Qian Hao, Xiang Zhou

**Affiliations:** 1 Fudan University Shanghai Cancer Center and Institutes of Biomedical Sciences, Fudan University, Shanghai 200032, China; 2 Department of Oncology, Shanghai Medical College, Fudan University, Shanghai 200032, China; 3 Key Laboratory of Breast Cancer in Shanghai, Fudan University Shanghai Cancer Center, Fudan University, Shanghai 200032, China; 4 Shanghai Key Laboratory of Medical Epigenetics, International Co-laboratory of Medical Epigenetics and Metabolism (Ministry of Science and Technology), Institutes of Biomedical Sciences, Fudan University, Shanghai 200032, China

The RNA component of mitochondrial RNA-processing endoribonuclease (RMRP) was first described as an entity that cleaved mitochondrial RNA at a priming site of mitochondrial DNA replication. This long noncoding RNA (lncRNA) was encoded by an evolutionarily conserved nuclear gene that has been characterized in many species, including human, mouse, cattle, zebrafish, toad, and yeast. It was soon learned that RMRP as a component of the RNase MRP complex was also located in the nucleus, and was critical for ribosomal RNA (rRNA) processing, particularly the biosynthesis of 5.8S rRNAs. In 2001, the first evidence of the role of RMRP in the pathogenesis of human disorders was reported, involving mutations in the *RMRP* gene, which caused cartilage-hair hypoplasia (CHH)^1^, a recessively inherited developmental disorder characterized by short stature, hypoplastic anemia, defective immunity, and predisposition to several cancers. Later studies suggested that mutations or underexpressions of RMRP were associated with cell growth arrest and developmental abnormalities in various organisms. Consistent with these findings, growing evidence has recently reported the tumor-promoting function of RMRP, which is essential for cancer cell survival and propagation, and which will be discussed below.

## Involvement of RMRP in cancer

The association of RMRP with cancer was first described in CHH patients with significantly increased risks of several types of cancers, especially lymphomas^1^. RMRP expression was upregulated, probably through the YAP/β-catenin transcription complex, in colorectal and breast cancer tissues, when compared with normal tissues. Recurrent mutations in the *RMRP* promoter may enhance recruitment of some transcription activators leading to increased RMRP expression and breast cancer progression^2^. Rare somatic mutations in the promoter region were also identified in leukemia, myeloma, gastric carcinoma, colorectal cancer (CRC), and sarcoma, suggesting that a low prevalence of *RMRP* promoter mutations could be a common feature of both solid and hematological malignancies. RMRP expression is elevated in non-small cell lung cancer tissues, when compared with matched adjacent normal tissues, and its higher expression is associated with higher tumor node metastasis stages and predictions of poor patient survival. It is also expressed at a higher level in lung cancer cell lines, such as A549 and H1299, when compared with normal epithelial cell lines. Multi-gene panels have been developed to assess biomarkers, including RMRP in plasma, for the early detection of lung cancer. In addition, RMRP is overexpressed in hepatocellular carcinoma (HCC), bladder cancer, papillary thyroid cancer, glioma, neuroblastoma, cholangiocarcinoma, and multiple myeloma, and the higher level of RMRP in tumors is associated with cancer progression and an unfavorable prognosis.

Our group recently examined the expression of RMRP in CRC using RT-qPCR and RNA *in situ* hybridization in two independent cohorts of patients^3^. We showed that RMRP expression was significantly upregulated in CRC samples, when compared with noncancerous tissues. Both univariate and multivariate analyses and Kaplan-Meier survival analysis revealed that higher expression of RMRP predicted worse prognoses in CRC patients^3^. In a later study, we further reported that RMRP expression was upregulated in various human cancers, when compared with normal tissues, and upregulation of RMRP was significantly associated with unfavorable prognoses in breast cancer, head and neck carcinoma, lung adenocarcinoma, pancreatic ductal adenocarcinoma, stomach adenocarcinoma, and uterine corpus endometrial carcinoma^4^. However, a few studies have reported that RMRP may be downregulated in cancers and may be associated with a favorable prognosis, thereby suggesting that RMRP may have divergent roles in the context of different pathological or stressful conditions. Taken together, overexpression of RMRP is common in most human cancers, and is associated with malignant progression and unfavorable prognoses, suggesting that RMRP is an oncogenic lncRNA.

## Regulation of RMRP expression by various stress signals

**Figure 1:**
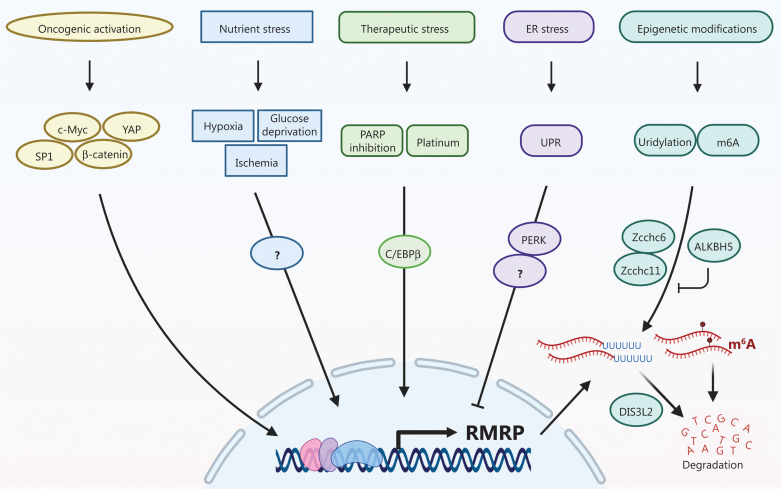
The expression of the RNA component of mitochondrial RNA-processing endoribonuclease (RMRP) is regulated in response to a variety of cellular stresses.

Although RMRP, as an rRNA biosynthesis-associated lncRNA, is expressed at relatively high levels in different tissues and species, there are still many mechanisms that control the expression of RMRP at an appropriate level in response to stress signals (**Figure 1**). RMRP can act as a nutrient sensor that is upregulated under conditions of hypoxia or ischemia. For example, RMRP expression is induced in HeLa cells and oral carcinoma cells under hypoxic conditions or when treated with the hypoxia-mimetic agent, cobalt chloride. Additionally, the levels of RMRP are elevated in neural cells and cardiomyocytes in cell culture systems or in ischemic mouse models during conditions of hypoxia or oxygen-glucose deprivation. However, the detailed molecular basis of how RMRP expression is modulated during nutrient depletion remains to be investigated.

The expression of RMRP is upregulated in response to various anti-cancer therapies. Our recent study revealed that poly(ADP-ribose) polymerase (PARP) inhibitors induced the expression of RMRP by the transcription factor, C/EBPβ. Consistently, we showed that expression of C/EBPβ was positively correlated with RMRP levels and was associated with poor prognoses in CRC patients^3^. Furthermore, RMRP expression may be elevated in cisplatin-resistant ovarian cancer cells, probably also through activation of C/EBPβ. Several other transcription factors, such as YAP/β-catenin, c-Myc, and SP1, have also been found to activate RMRP expression, which in part explains why this lncRNA is upregulated in several cancers.

In addition, RMRP expression is regulated by endoplasmic reticulum (ER) stress. ER stress is induced by perturbation of calcium ion homeostasis, inhibition of protein glycosylation, oxidative stress, and accumulation of misfolded proteins, all of which impair ER function. In response to ER stress, the ER initiates several signaling pathways, such as the unfolded protein response (UPR), leading either to cell survival or cell death. The UPR is characterized by activation of the protein kinase R (PKR)-like ER kinase (PERK). Recently, RMRP was identified as a PERK-downregulated gene using RNA-sequencing analysis, which showed that tunicamycin-induced ER stress led to a decrease in RMRP expression, eventually resulting in caspase activation and HCC cell apoptosis, while knockdown of PERK restored the level of RMRP. However, further studies are needed to elucidate how PERK inhibits RMRP expression under ER stress.

Moreover, posttranscriptional modifications contribute to RMRP expression by modulating its RNA turnover. The terminal uridylyl transferases, Zcchc6 and Zcchc11, mediate uridylation at the 3′ end of RMRP, which in turn results in degradation by the 3′-5′ exonuclease, DIS3L2. The 3′-uridylated RMRP was found to be accumulated and stabilized in the cytoplasm of DIS3L2-depleted cells, which might be a potential mechanism for Wilm’s tumorigenesis caused by germline mutations in *DIS3L2*. RMRP may also undergo N6-methyladenosine (m^6^A) modification, resulting in its rapid turnover, while the m^6^A demethylase, ALKBH5, increases RMRP expression through demethylation of the latter, which leads to lung cancer growth *in vitro* and *in vivo*.

Overall, RMRP can be regarded as a multi-stress sensor whose expression is controlled by diverse stress signals, including nutrient depletion, therapeutic stress, oncogene activation, ER stress, and epigenetic alterations.

## RMRP promotes cancer development as a microRNA sponge

**Figure 2:**
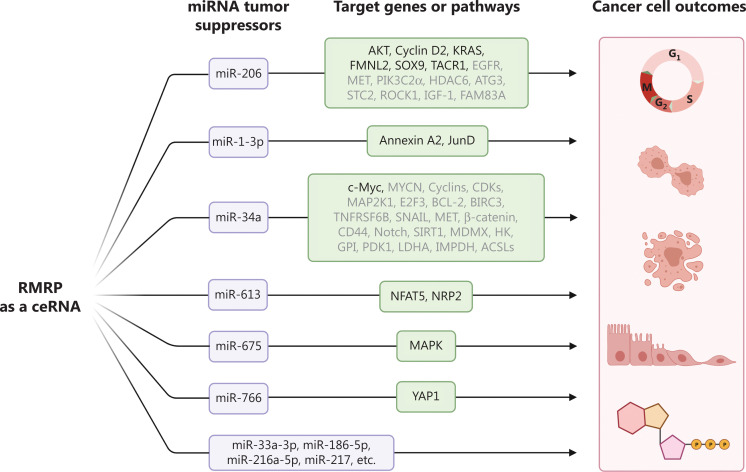
The RNA component of mitochondrial RNA-processing endoribonuclease (RMRP) promotes cancer cell growth and proliferation, inhibits apoptosis, drives the epithelial-mesenchymal transition and metastasis, and regulates energy metabolism by competitively interacting with numerous miRNA tumor suppressors.

It has been established that a major function of lncRNA involves competitive endogenous RNAs (ceRNAs) that derepress the expression of microRNA (miRNA) target genes by competitively associating with miRNAs. Emerging evidence suggests that RMRP promotes tumor growth and progression by a ceRNA mechanism (**Figure 2**)^5^. The myomiR family member, miR-206, is a multi-faceted miRNA that is involved in skeletal muscle development and pathology, Alzheimer’s disease, chronic obstructive pulmonary disease, and different types of cancer. RMRP was found to sequester miR-206 to release transcripts of cyclin D2, resulting in gastric cancer cell cycle progression and increased tumor growth. This finding is consistent with several studies on CHH patients, as mutations in the *RMRP* gene result in cell cycle abnormalities in lymphocytes, leukocytes, chondrocytes, and fibroblasts of patients with CHH. Because miR-206 has been shown to inactivate multiple oncogenic signal transduction pathways in cancer, it is not surprising that RMRP is also able to regulate these pathways by counteracting miR-206. We recently reported that RMRP promoted breast cancer cell growth and migration by activating the AKT pathway^4^. RMRP prevents miR-206 from directly targeting AKT mRNA for degradation by competitively binding to miR-206. More importantly, overexpression of RMRP aborts miR-206-induced repression of breast cancer cell growth and migration, while ablation of AKT completely restores RMRP-induced malignant phenotypes^4^. In agreement with our study, miR-206 was also shown to indirectly repress AKT activity by targeting EGFR, MET, PIK3C2α, HDAC6, ATG3, STC2, ROCK1, IGF-1, or FAM83A. Additionally, RMRP sequesters miR-206 to induce the expression of KRAS, FMNL2, SOX9, and TACR1, resulting in activation of various tumor-promoting signaling pathways and subsequent cancer progression. Another myomiR member, miR-1-3p, sharing the same seed sequence with miR-206, is also trapped by RMRP. It was found that RMRP upregulated expression of Annexin A2 and the JunD proto-oncogene, which supported cancer growth and therapeutic resistance, by arresting and reducing the level of miR-1-3p. Sequestration of the two myomiRs contributes to RMRP-mediated tumor growth and metastasis, and may partially explain why CHH patients show defects in cell cycle progression and cartilage development.

Another important example of RMRP-targeted miRNAs is miR-34a-5p, also known as miR-34a, which is transcriptionally activated by the tumor suppressor p53. The miR-34a was found to control cell cycle progression, promote apoptosis, suppress the epithelial-mesenchymal transition (EMT) and metastasis, and reprogram cancer metabolism by inhibiting the expression of a large number of genes. The finding that RMRP acts as a sponge for miR-34a implies that this lncRNA may regulate myriad cellular processes during cancer development by inactivation of miR-34a, which will be an important topic of investigation in future studies. Intriguingly, miR-34a inhibits SIRT1, which mediates deacetylation of the p53 protein and MDMX, which promotes p53 ubiquitination in cooperation with MDM2, implying that RMRP might repress p53 in miR-34a highly-expressing cancer cells.

Besides the abovementioned miRNAs, several others have been found to act as RMRP targets. It was reported that depletion of RMRP suppressed cholangiocarcinoma cell growth *in vitro* and *in vivo* by upregulating a panel of miRNAs, such as miR-33a-3p, miR-186-5p, miR-216a-5p, and miR-217. Specifically, RMRP enhances cholangiocarcinoma cell proliferation and colony forming ability by inhibiting miR-217 expression. The level of miR-217 was consistently negatively correlated with RMRP in cholangiocarcinoma tissues. RMRP promotes cancer cell proliferation and migration by inhibiting miR-613, to induce expression of NFAT5 or NRP2 in HCC, lung cancer, and esophageal squamous cell carcinoma. It also elevates MAPK expression by repressing miR-675 in papillary thyroid cancer. Moreover, it was recently found to regulate the miR-766/YAP1 axis, resulting in triple-negative breast cancer progression. Together, like many other lncRNAs, RMRP can function as a ceRNA, which entraps various tumor suppressive miRNAs to promote cancer development.

## RMRP triggers tumor resistance to PARP inhibitors by inhibiting p53

Inhibition of PARP activity prevents single-strand DNA lesions that are eventually converted to double-strand breaks. PARP inhibitors are therefore widely used to treat tumors with homologous recombination deficiencies, which is a DNA repair pathway for double-strand breaks, caused by mutations in BRCA1/2 or other DNA damage repair (DDR) genes^6,7^. It is believed that tumors harboring the p53 mutation or deficiency are more sensitive to PARP inhibitors. Wild-type p53 is a tumor suppressor that is able to transcriptionally activate several cell cycle and DDR genes. It is likely that p53 mutations or deficiencies impair the DDR pathway, mediating tumor vulnerability to additional DNA insults caused by PARP inhibitors. Another possible mechanism involves missense mutant p53 proteins, which can increase PARP1 association with chromatins and facilitate PARP1-dependent aberrant DNA repair, leading to a strong tumor dependency of the PARP1/mutant p53 repairing complex^8,9^. In contrast, several studies also suggest that the presence of wild-type p53 may increase tumor sensitivity to PARP inhibition, as PARP inhibitors can induce p53-dependent cell death^10,11^. Together, these studies imply that p53-dependent DDR and apoptotic pathways play distinct roles in PARP inhibition-associated therapy, leading to an intriguing question involving what mechanism determines p53 responses to PARP inhibitors.

**Figure 3:**
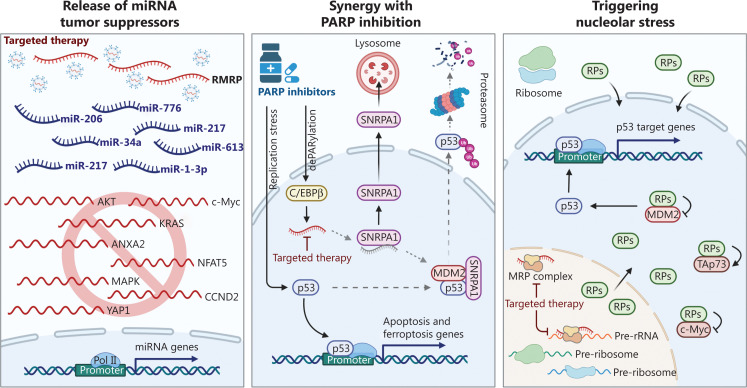
Targeting the RNA component of mitochondrial RNA-processing endoribonuclease (RMRP) as an anti-cancer strategy. Inhibition of RMRP by exosome-mediated delivery of antisense oligonucleotides leads to derepression of miRNA tumor suppressors (left panel), complete activation of the p53 pathway (middle panel), and nucleolar stress (right panel).

In our recent study, RMRP was shown to attenuate p53 activity in response to PARP inhibition, thus leading to CRC resistance to PARP inhibitors^3,12^. First, we found that RMRP was upregulated in CRC tissues, when compared with adjacent normal tissues, and a higher level of RMRP was associated with a worse prognosis, which prompted us to identify the role and mechanism of action of this lncRNA in CRC. Using transcriptomics analysis, the p53 pathway was identified to be most prominently activated upon RMRP knockout. Overexpression of RMRP promotes, whereas ablation of RMRP represses, CRC cell growth *in vitro* and *in vivo* by regulating p53 activity. Mechanistically, we identified an uncharacterized signaling pathway responsible for RMRP-mediated p53 inhibition. Small nuclear ribonucleoprotein polypeptide A’ (SNRPA1), a subunit of the spliceosome complex, is associated with progression of several types of cancers. Our study revealed that RMRP directly interacted with SNRPA1 to detain the latter in the nucleus and, thereby, prevent lysosomal degradation of SNRPA1 through chaperone-mediated autophagy. Nuclear SNRPA1 then binds to p53 and enhances MDM2-induced p53 degradation. More intriguingly, PARP inhibitors are able to induce RMRP expression by derepressing the transcription factor, C/EBPβ, as PARP1-mediated PARylation impedes C/EBPβ binding to its target gene promoters^13^. It is therefore proposed that activation of the C/EBPβ-RMRP-SNRPA1 axis results in p53 inactivation and tumor resistance to PARP inhibition. Further supporting evidence suggests that depletion of RMRP significantly boosts p53 activation and cancer cell elimination caused by olaparib-associated therapies^3^. Our study suggested that upregulation of RMRP decreased p53 activation upon PARP inhibition, leading to cell cycle arrest and DNA repair, while a deficiency in the C/EBPβ-RMRP-SNRPA1 axis led to full activation of p53, resulting in p53-dependent cell death (**Figure 3**)^3,12^.

## Future perspectives: RMRP as a potential therapeutic target in cancer

There is considerable evidence supporting RMRP as a therapeutic target in cancer therapy (**Figure 3**). First, RMRP is highly expressed in many types of human cancers, and a higher level of RMRP predicts a worse prognosis. In addition, RMRP sequesters many miRNA tumor suppressors, resulting in accelerated cancer cell proliferation and metastasis. Moreover, RMRP compromises olaparib-induced p53 activation, thus conferring tumor resistance to PARP inhibition-associated therapies. In addition to those functions, RMRP may play a role in maintaining the integrity of the nucleolar architecture, because it is critical for biosynthesis and processing of pre-rRNAs^14^. It has long been observed that morphological changes in the nucleolus are reliable markers of cellular transformation. Enlarged nucleoli are found in various human cancers, as rapidly growing cancer cells require more active ribosome biogenesis, which mainly occurs in the nucleoli. Perturbation of multi-step ribosome biogenesis, namely nucleolar stress or ribosomal stress, triggers a series of downstream events, including the release of ribosome-free ribosomal proteins (RPs) to the nucleus, interaction of RPs with MDM2 and other proteins, and p53/TAp73 activation and c-Myc inhibition, eventually leading to cancer cell growth arrest and apoptosis^15,16^. Therefore, eliciting nucleolar stress by interrupting the RNA Pol I function is a potential therapeutic strategy for cancers^16^. Though highly possible, it is yet to be determined that inhibition of RMRP may also induce nucleolar stress by impairing rRNA production. Effectively targeting an RNA molecule, like RMRP, in patients is a challenge yet to be circumvented in the development of an anti-cancer approach. Fortunately, recent advances in nanoparticle- or engineered exosome-mediated delivery of antisense oligonucleotides (ASOs) provide a possible solution to target these “undruggable” ncRNA genes. Future research is therefore needed to develop new therapeutic methods of targeting RMRP in cancers expressing high levels of RMRP or in cancers resistant to PARP inhibitors.
